# Fear of Cancer Recurrence, Health Anxiety, Worry, and Uncertainty: A Scoping Review About Their Conceptualization and Measurement Within Breast Cancer Survivorship Research

**DOI:** 10.3389/fpsyg.2021.644932

**Published:** 2021-04-12

**Authors:** Christine Maheu, Mina Singh, Wing Lam Tock, Asli Eyrenci, Jacqueline Galica, Maude Hébert, Francesca Frati, Tania Estapé

**Affiliations:** ^1^Faculty of Medicine and Health Sciences, Ingram School of Nursing, McGill University, Montréal, QC, Canada; ^2^Faculty of Health, School of Nursing, York University, Toronto, ON, Canada; ^3^Department of Psychology, Faculty of Humanities and Social Sciences, Maltepe University, Istanbul, Turkey; ^4^Faculty of Health Sciences, School of Nursing, Queen's University, Kingston, ON, Canada; ^5^Département des Sciences Infirmières, Université du Québec à Trois-Rivières, Trois-Rivières, QC, Canada; ^6^Schulich Library of Physical Sciences, Life Sciences, and Engineering, McGill University, Montréal, QC, Canada; ^7^Psychosocial Oncology Department, Fundació per l'Educació i la Formació en Càncer (FEFOC) Fundació, Barcelona, Spain

**Keywords:** fear of cancer recurrence, health anxiety, uncertainty, worry, conceptualization, measurement, scoping review, cancer survivorship

## Abstract

**Objective:** Fear of Cancer Recurrence (FCR), Health Anxiety (HA), worry, and uncertainty in illness are psychological concerns commonly faced by cancer patients. In survivorship research, these similar, yet different constructs are frequently used interchangeably and multiple instruments are used in to measure them. The lack of clear and consistent conceptualization and measurement can lead to diverse or contradictory interpretations. The purpose of this scoping review was to review, compare, and analyze the current conceptualization and measurements used for FCR, HA, worry, and uncertainty in the breast cancer survivorship literature to improve research and practice.

**Inclusion Criteria:** We considered quantitative, qualitative, and mixed methods studies of breast cancer survivors that examined FCR, HA, worry, or uncertainty in illness as a main topic and included a definition or assessment of the constructs.

**Methods and Analysis:** The six-staged framework was used to guide the scoping review process. Searches of PubMed, CINAHL, and PsycINFO databases were conducted. The principle-based qualitative analysis and simultaneous content analysis procedures were employed to synthesize and map the findings.

**Findings:** After duplicate removal, the search revealed 3,299 articles, of which 82 studies met the inclusion criteria. Several critical attributes overlapped the four constructs, for example, all were triggered by internal somatic and external cues. However, several unique attributes were found (e.g., a sense of loss of security in the body is observed only among survivors experiencing FCR). Overall, findings showed that FCR and uncertainty in illness are more likely to be triggered by cancer-specific factors, while worry and HA have more trait-like in terms of characteristics, theoretical features, and correlates. We found that the measures used to assess each construct were on par with their intended constructs. Eighteen approaches were used to measure FCR, 15 for HA, 8 for worry, and 4 for uncertainty.

**Conclusion:** While consensus on the conceptualization and measurement of the four constructs has not yet been reached, this scoping review identifies key similarities and differences to aid in their selection and measurement. Considering the observed overlap between the four studied constructs, further research delineating the unique attributes for each construct is warranted.

## Introduction

The psychosocial impact of a cancer diagnosis is increasingly recognized as a significant clinical issue. The most extensively assessed constructs in psycho-oncology are Fear of Cancer Recurrence (FCR), Health Anxiety (HA), worry, and uncertainty in illness (Miller, 2012; Costa et al., 2016; Lebel et al., 2016a; Strout et al., 2018; Mutsaers et al., 2019). Yet despite the rapid proliferation of psycho-oncology research, the standardized conceptualization and measurement of these four core constructs have not yet been established and they are frequently used interchangeably (Bradford et al., 2013; Jones et al., 2014; Butow et al., 2019). Further, although associations between these constructs have been established (Fink et al., 2004; Moye et al., 2014), their use as proxies for one another is not empirically supported (Consedine et al., 2004; Hirai et al., 2008). In part, this practice reflects a lack of clarity on their categorical distinctions. For example, health anxiety has been used to describe the fear and worry in response to living with a chronic illness (Asmundson et al., 2010; Lebel et al., 2020); similarly, fear and worry have also been used to characterize FCR (Lebel et al., 2016b). To further muddle the distinctions among these four constructs, uncertainty in illness has been defined as the phenomenological experience of anxiety arising from unpredictable real or unreal health issues (Grupe and Nitschke, 2013), using terms often found in the other studied constructs. While FCR has been proposed as a unique psychosocial issue (Mutsaers et al., 2019), its clinical characteristics overlap with the established diagnostic criteria for HA and are also related to worry (Commons et al., 2016).

In an attempt to clarify the nature and characteristics of FCR, a group of content experts met in 2015 to formulate a consensual definition of FCR using the Delphi method (Lebel et al., 2016b). The researchers determined that FCR is a multidimensional construct encompassing triggers, emotions, thoughts, physiological reactions, and coping strategies. They described a broad-spectrum definition of FCR as: “Fear, worry, or concern about cancer returning or progressing.” (Lebel et al., 2016b). Conversely, Costa et al. proposed that although FCR is commonly recognized as a multidimensional construct, further research was required to determine the core construct of FCR, which in turn would facilitate the development of shorter, more easily decipherable FCR measures (Costa et al., 2016).

Beyond cancer and FCR, the constructs of uncertainty, worry, and HA have also been defined and applied to a variety of health, illness, and other psycho-social contexts. Mishel's widely used Theory of Uncertainty in Illness defined health and illness-related uncertainty as “the inability to determine the meaning of illness-related events, and accurately anticipate or predict health outcomes (Mishel, 1988).” In their conceptualization, Dugas et al. observe that in worry, “counterproductive beliefs, appraisals, and expectations may interfere with the individual's ability to use problem-solving behavioral skills (Dugas et al., 1995).” Lastly, HA has been conventionally conceptualized as “a multifaceted phenomenon consisting of distressing emotions, physiological arousal and associated bodily sensations, thoughts and images of danger, and avoidance and other defensive behaviors (Lang, 1985; Bradford et al., 2013).” In this approach, levels of health anxiety can vary along a continuum: on one end, HA is a healthy response and a motivation for performing positive health behaviors, whereas, on the opposite end, HA becomes pathological and dysfunctional.

In the area of measurement and assessment, there are also significant overlaps. For example, the Impact of Event Scale (Sundin and Horowitz, 2002) has been used measure both cancer-related anxiety and FCR (Thewes et al., 2001; Custers et al., 2016). Its use in FCR measurement applied the scale's sub-items such as intrusive and unpleasant thoughts to the worry that cancer could come back (i.e., FCR). Added to the psychometric confusion or “noise,” some FCR instruments included items assessing other psychological constructs concerning cancer patients, such as anxiety and worry (Costa et al., 2016). In these ways, the use of the same instrument to measure multiple constructs, and the incorporation of multiple constructs within a single instrument further perpetuates the confusion of what tool is best to use to specifically measure each of FCR, worry, health anxiety, or uncertainty. Thus, both the conceptual distinctions between and the empirical clinical measurement of these constructs remain unclear.

While consensus on these distinctions has yet to be achieved, researchers do agree that distinct constructs and standardized definitions and measurements are required to effectively apply them to clinical practice and research (Costa et al., 2016; Lebel et al., 2016b; Maheu and Galica, 2018; Butow et al., 2019). A common understanding of these constructs will help clinicians and researchers to target their care and interventions to specific cancer survivors' psychosocial needs and mental health outcomes. The identification of the core components and characteristics of the four constructs can improve measurement and screening capacity, and intervention development and implementation in the field of psycho-oncology.

The purpose of this scoping review, therefore, was to provide an overview of current use to conceptualize (i.e., characteristics, theoretical features, triggers, and correlates) and measure FCR, HA, worry and uncertainty as applied within a frequently occurring type of cancer, breast cancer. Our aim was to compare similarities and differences in how constructs have been used in research and map out existing evidence to contribute to the clarification of the application and measurement of these constructs in survivorship research, practice, and the development of interventions. The results of this review will guide future survivorship research relevant to the four different psychological concerns.

## Method

The scoping review method applies a rigorous literature review process to investigate a body of literature on emerging or diverse topics, map out key constructs, clarify their definitions, and establish conceptual boundaries (Peters et al., 2020). Scoping review results are typically used to inform clinical decision-making and practice and provide direction for future research (Peters et al., 2020). In this review, we employed the Joanna Briggs Institute's (JBI) scoping review methodological framework, originally developed by Arksey and O'Malley (2005) and refined by Levac et al. (2010). Its six stages include the identification of the research question and relevant studies; study selection; data charting; collating, summarizing, and reporting results; and consultation (Arksey and O'Malley, 2005). Our scoping review was conducted in accordance with the Preferred Reporting Items for Systematic Reviews and Meta-Analyses Extension for Scoping Reviews (PRISMA-ScR) checklist (Tricco et al., 2018) (i.e., refer to Supplementary File 1 for the completed checklist). Critical appraisal and risk of bias assessment of studies are not mandatory in scoping reviews (Peters et al., 2020) and so these steps were not conducted. The scoping review protocol was registered with the Open Science Framework (OSF): (https://osf.io/78hxj/).

### Stage I: Research Question and Objectives

Our research question was: “*What are the similarities and differences in the conceptualization (i.e., characteristics, theoretical features, triggers, and correlates) and measurement of the following constructs: FCR, HA, worry, and uncertainty in breast cancer survivor research*?” A sub-question was: “*How are HA, worry, and uncertainty similar to, or different from FCR?”* Our objectives were to summarize the conceptualization and empirical measurement of the four constructs in the breast cancer survivorship literature and provide recommendations to guide future survivorship interventions and research.

### Stage II: Relevant Literature Identification

The search strategy followed the JBI iterative three-step process (Aromataris and Munn, 2020a): an initial search of the selected database using pre-specified keywords; a second thorough search across all included databases; and a final review of the reference lists of included studies to identify any missing studies. For the second step, only relevant articles from the initial screening were analyzed to inform the final search (Morris et al., 2016; Aromataris and Munn, 2020b). The academic librarian (FF) for the McGill Ingram School of Nursing and Affiliated Health Institution Libraries conducted the step one search in Ovid MEDLINE. These results were peer-reviewed by a second librarian (see Appendix A: Search Strategies); the revised search was conducted on March 11, 2020, in Ovid MEDLINE(R) ALL 1,946 onwards and CINAHL Plus with FullText (EBSCO). FF exported the search results from the Medline and Cinahl searches into EndNote X9 (Clarivate, PA, USA). Two reviewers (MS & WLT) screened results with the inclusion criteria; FF analyzed results using Yale's Medical Subject Headings [MeSH] analyzer to identify additional keywords and MeSH subject headings. The revised search was conducted on May 22, 2020, in Medline and Cinahl and was translated into APA PsycInfo 1967 onwards (second step of the search process). FF exported all results into EndNote and removed duplicates and articles already screened using a simplified method recommended by Bramer et al. (2016). Two reviewers (MS & WLT) conducted the second round of screening of results from all databases. For the third step, these reviewers screened the reference list of all relevant studies for additional relevant studies and used Google Scholar, Scopus, and the related article feature of Pubmed to identify citing articles of relevant studies. Two reviewers (AE & CM) screened these citing articles for inclusion. Reviewers hand-searched relevant therapeutic and special topic journals, contacting subject experts as needed.

### Stage III: Study Selection

The team consolidated search results, removing duplicates, using Endnote and Covidence. Two reviewers (MS & WLT) screened article titles and abstracts to exclude those that did not meet the eligibility criteria. Disagreements about article eligibility were resolved by arbitration of a third reviewer (MH). For those fulfilling the eligibility criteria, the full article was retrieved. Two team members (AE & WLT) screened the full text of the articles, again excluding those not meeting the eligibility criteria. Eligibility disagreements were discussed between the two reviewers until consensus was reached or again, were arbitrated by a third reviewer (MH).

**Figure 1:**
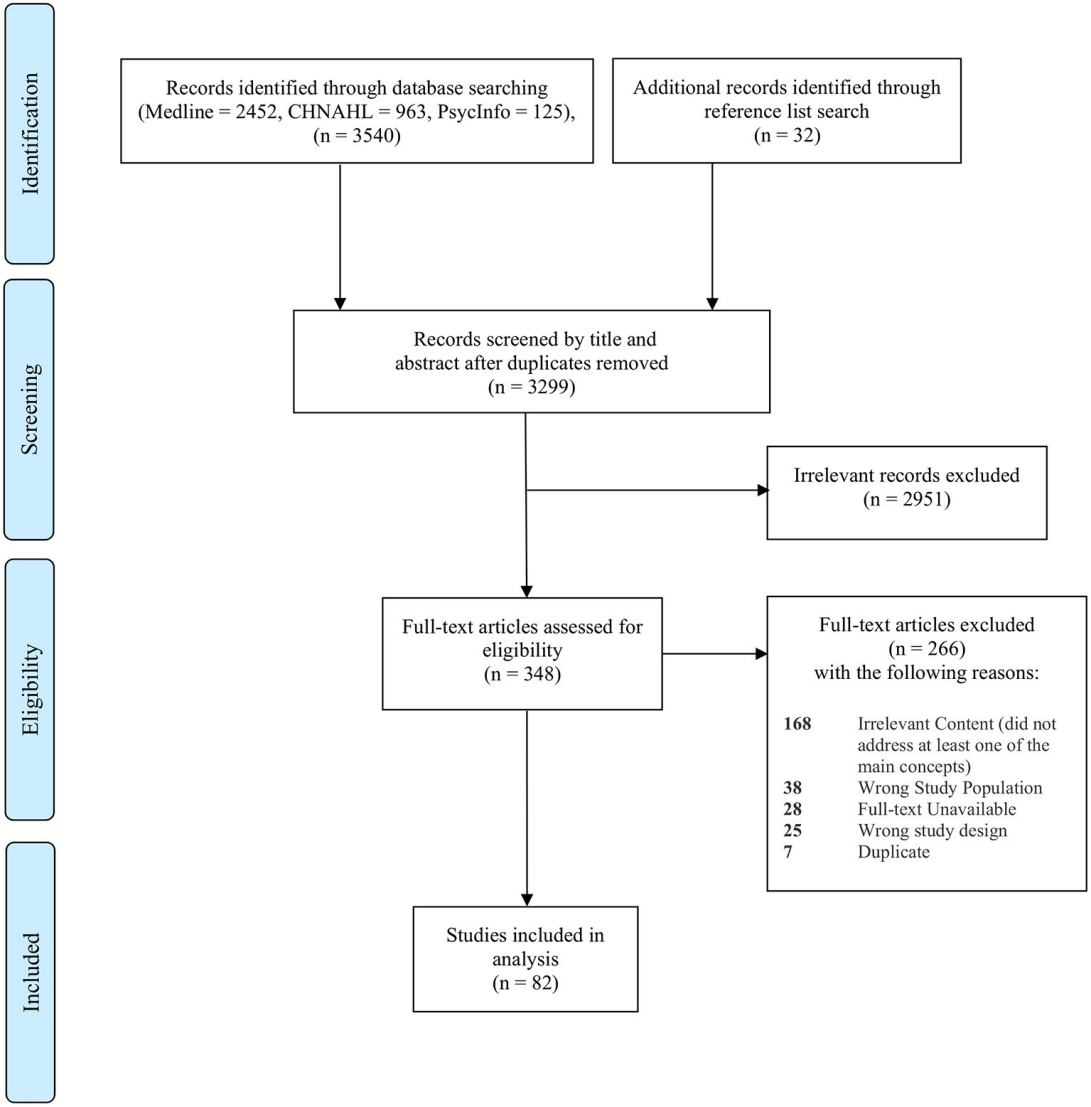
Preferred Reporting Items for Systematic Reviews and Meta-Analyses Extension for Scoping Reviews (PRISMA-ScR) selection of sources of evidence flow diagram.

The primary inclusion-exclusion criteria were summarized in Table 1. Throughout the study selection process, the inclusion and exclusion criteria were refined iteratively, as the reviewers calibrated the threshold for inclusion and exclusion through discussion and consensus and with input from the entire research team. Finally, in accordance with PRISMA-ScR guidance, the study selection process with detailed reasons for study exclusions is presented in a PRISMA-ScR flow diagram (see Figure 1).

**Table 1:** Study inclusion and exclusion criteria.

**Study characteristics**	**Inclusion criteria**	**Exclusion criteria**
Design	A primary quantitative or qualitative research. We included only the primary research if a secondary analysis of the same set of data was available.	Case report, protocols, reviews of the literature, and conference proceedings
Publication types	Peer-reviewed journal; the full article describing the research was available in English.	Commentaries, books, book reviews, letters to the editor, theses, opinion papers, abstracts without full-text, or articles without an English full-text.
Participants	Participants must involve women with stage 0–3 breast cancer, ductal carcinoma *in situ* and lobular carcinoma *in situ* are considered stage 0 breast cancer; participants must have completed initial treatment (chemotherapy or surgery) but could be on hormone therapy	The participants in the study had metastatic or recurrent cancer; participants were undergoing genetic testing or counseling, as this was considered to be a form of treatment.
Study concepts	Included Fear of Cancer Recurrence (FCR), Health Anxiety (HA), Worry or Uncertainty as a major concept. Fear of progression (FoP) is used interchangeably with FCR; therefore, we included the term FoP in our search and selection process.	The concepts under study included fear of disfigurement, fear of having children, fear of returning to society, etc.

### Stage IV: Data Extraction (Data Charting)

Based on the preliminary scoping phase, the team developed a data extraction framework that defined 18 categories (see Appendix B “Data Extraction Instrument”). This instrument was built into the Covidence extraction tool. Alongside standard bibliographical information (i.e., authors, title, journal, and year of publication), the design of the study, study setting, and study purposes were extracted. For each study, characteristics of the study populations, including age, marital status, ethnicity, stage of cancer diagnosis, time since the end of treatment/ time since diagnosis were also documented. For studies that validated a psychometric scale or instrument to measure one of the four constructs, we included studies with participants of mixed cancer diagnosis; in such cases, we documented the percentage of breast cancer patients if the participants consisted of mixed samples of cancer survivors. From validation studies, we also extracted information on the research question, including the definition and conceptualization framework, the assessment tool (i.e., measurements/scales/questionnaires/interview questions), details of psychometric validation of the tool, and the characteristics of any of the four constructs described in the results and discussion sections.

Questions arising during the data extraction stage were discussed by the team and disagreements were resolved through team consultations. When necessary, the categories were modified, and the data extraction instrument was revised accordingly. Four members of the team (two pairs: TE & JG, and CM & WLT) independently extracted data from each study using the data extraction feature in Covidence. To ensure inter-rater reliability, extracted data were compared. Any discrepancies in extracted data were discussed between the reviewers until consensus was reached or by arbitration of a third reviewer, as required.

### Stage V: Data Analysis and Synthesis

We developed a structured approach to synthesize and collate review data by modifying and combining the principle-based qualitative analysis of Morse and Field (1995) and simultaneous content analysis procedures (Haase et al., 1993). Notably, we followed Morse and Field (1995) in aligning the purpose of the analysis with the complexity of the constructs, expanding structural features of attributes as needed. We also drew from Haase et al. (1993) simultaneous concept analysis process and we used an iterative process of examining relationships across constructs using consensus groups.

To address our research questions, two major attribute categories were formed: (1) conceptualization and (2) measurements. In order to clarify the characterization of the four constructs, we have incorporated guidance from principle-based content analysis procedure (Penrod and Hupcey, 2005) and established an expanded classification of the first category, conceptualization, resulting in 4 sub-categories: (a) characteristics, (b) theoretical features, (c) triggers, (d) correlates. We developed a standardized coding guideline (see Table 2) defining each of the critical attribute categories and subcategories to guide the coding process. For this review, we defined “Characteristics” as the description or statement containing defining features that aid in determining which phenomena match the construct and usually followed the key phrases such as “defined as” or “described as.” “Theoretical features” we defined as the presence of specific features or indications in the theoretical model that aid in understanding the construct. We defined “Triggers” as the events or antecedents that present prior to the specific phenomenon of the construct. Finally, we defined “Correlates” as factors shown to have an association with the given construct.

**Table 2:** Critical attribute categories and coding guidance for categorizing the four psychological constructs.

**Critical attribute category**	**Critical attribute definition and coding guidance**	**Examples^a^**
**Conceptualization**	Conceptualization of the four constructs is demonstrated in the following four ways: • **Characteristics**. The description or statement containing defining indicators that aid in determining which phenomena match the construct and which do not. • **Theoretical features**. The presence of specific features or indications in the theoretical model/ framework that aid in understanding the construct. • **Triggers**. The events or antecedents that are presented prior to the specific phenomenon of the construct. • **Correlates**. The factors that have been shown to have an association with the construct. Only correlates that are descriptions of primary study findings will be coded. The direction of the relationship will not be described.	**Characteristics** • “FCR is defined as the **“fear or worry that the cancer will return or progress in the same organ or in another part of the body**.” [13] **Theoretical features** • “According to Leventhal's self-regulation model of illness, an individual's level of FCR is determined by his/her **illness representation through cognition and emotional processing**.” [7] **Triggers** • “Uncertainty is generated when **components of illness or treatment possess the characteristics of inconsistency, randomness, complexity, unpredictability, and lack of information** in situations of importance to the individual” [41] **Correlates** • “**Social constraints** demonstrated a significant indirect effect on FCR through the mechanism of cognitive processing.” [69] • “Anxiety was positively associated with **depression and symptom severity**.” [8]
**Measurements**	• Instruments designed to determine the quantity of a variable within the concepts (i.e., **questionnaires, inventories, scales, surveys, and interviews**). Both quantitative and qualitative measurements will be documented.	• “Health anxiety is measured using the **Short Health Anxiety Inventory (SHAI)**, it demonstrates good reliability and validity and discriminates between individuals with and without hypochondriasis.” [46] • “The **Mishel Uncertainty in Illness Inventory Scale-Community version (MUII)** is used to measure uncertainty. The MUII is a 33-item scale that measures an individual's clarity, understanding, and certainty regarding their illness.” [63] • “FCR was assessed in a **semi structured interviews:** women described their thoughts and feelings regarding the possibility of recurrence, the nature of their fears, the circumstances under which their fears were most salient (i.e., what triggered their fears), and their efforts to cope with those fears.” [77]

a *refer to Supplementary File 2 for a complete listing of the references*.

The extracted data (i.e., refer to Supplementary File 2 for the extracted data and a complete listing of the references) were analyzed according to the coding guideline. The critical attributes of the four constructs were then transferred into summaries across studies using *in vivo* coding (using a word or short phrase taken from that section of the data). Every textual description of a conceptualization was categorized into a single subcategory. To facilitate the consistency of categorization, one team member (WLT) carried out the initial coding procedures, and the resulting construct matrices were reviewed independently by two team members (CM & MS) (see Tables 3, 4 for the two construct matrices, with articles numbers reflected in Supplementary File 2). The review team met bi-weekly to discuss the process and results of the data synthesis, validating the synthesis methodology, auditing the decision-making trail, and providing feedback on the analysis results. Modifications to the coding procedures were made to reflect the re-examination of the preliminary results. The study characteristics and coded evidence were summarized using both qualitative and quantitative techniques and were then tabulated in an aggregate and visual form (i.e., matrix tables and bubble graphs). The tabulated summary was elaborated narratively, addressing the research questions and scoping review objectives.

**Table 3:** Conceptualization: number and prevalence of thematically derived critical attributes for the four constructs: article numbers reflected in Supplementary Material.

**Critical Attribute: conceptualization subcategories**	**Constructs^a^**			
	**Fear of Cancer Recurrence (FCR) *n* = 73**	**Health Anxiety (HA) *n* = 38**	**Worry (W) *n* = 11**	**Uncertainty (U) *n* = 15**
**Characteristics** **=** **13**				
The concern that cancer will come back or progress	[3] [6] [15] [16] [30] [40] [41] [42] [46] [49] [56] [57] [58] [60] [63] [66] [67] [69] [70] [71] [72] [73] [74] [78] [81] [82] [76]	—	[61]	—
A type of cancer-related worry	[17] [30] [32] [33] [34] [41] [49] [70] [79] [81]	—	[17] [41] [61]	—
A subset of anxiety	[13] [30] [40] [46] [49] [51] [54] [67]	—	—	—
Trait anxiety	—	[6] [40] [48] [62]	—	—
State anxiety	—	[6] [40] [48] [62]	—	—
A symptomatic consequence of anxiety	—	—	[17] [54]	—
A type of emotional reaction	—	—	[61]	—
The inability to determine the meaning or outcome of the illness	—	—	—	[10] [25] [26] [52]
Being in doubt, being undecided, perceptions of vagueness	—	—	—	[29] [68]
A state of liminality	—	—	—	[75]
A mismatch between one's expectation and the realistic world	—	—	—	[80]
A moderator between triggers and FCR	—	—	—	[41]
A trigger of FCR	—	—	—	[10] [12] [25] [26] [27] [41] [52] [55] [56] [80]
**Theoretical features** **=** **23**	**FCR**	**HA**	**Worry**	**Uncertainty**
Excessive seeking of professional advice for reassurance	[1] [2] [15] [21] [23] [35] [40] [41] [57] [58] [72] [76]	[49] [58]	[54]	[41]
Worry, rumination or intrusive thoughts	[3] [14] [35] [40] [51] [59] [63] [71] [72]	[73]	[10] [71]	—
Excessive personal checking behavior	[15] [27] [35] [41] [43] [46] [57] [71] [72] [75] [76]	[49] [58] [72]	[19]	—
Misinterpretation of neutral bodily symptoms	[15] [40] [46] [76]	[41]	[67]	—
Adoption of avoidance-oriented coping	[16] [30] [35] [40] [41] [51] [63] [65] [71] [72] [76]	[35] [58] [72]	[54]	—
Anxious preoccupations	[1] [3] [14] [15] [43]	[46]	—	—
Determined by illness representation	[7] [12] [24] [37] [44] [46] [65] [66] [81]	[38]	—	—
Increased vigilance to somatic sensations	[40] [46] [51]	[41]	—	—
Ongoing, persisting and stable over time	[4] [10] [22] [27] [33] [34] [57] [59] [60] [66] [72] [75] [76] [78]	—	[61]	—
Multidimensional	[9] [37] [42] [78] [82]	—	—	[9] [25] [26]
Realistic fear	[37] [43]	—	—	—
Excessive concern about the treatment adverse effects	[21] [27] [44] [64]	—	—	—
Extra reassurance serves to maintain patients' fear	[58] [73]	—	—	—
Loss of a sense of security in the body	[75]	—	—	—
Estimation of danger enhanced by threat-related stimuli	—	[13]	—	—
Unrealistic fear	—	[46] [54] [58] [72] [76]	—	—
Autonomic arousal	—	[6] [40] [62] [73]	—	—
A trend decreases over time	—	[23] [38]	—	—
Occurs among individuals without a medical problem	—	[46] [76]	—	—
Experience an anxiety/relief cycle	—	[1]	—	—
Self-focused attention	—	—	[71]	—
Perceptual state that existed on a continuum changes over time	—	—	—	[27] [29] [52] [55] [80]
Not feeling secure and safe from danger	—	—	—	[29]
**Triggers** **=** **14**	**FCR**	**HA**	**Worry**	**Uncertainty**
Internal (somatic) cues such as physical symptoms	[10] [12] [23] [24] [26] [27] [33] [44] [50] [54] [65] [66] [71] [79]	[30] [44]	[17] [19]	[27] [52] [55] [64]
External cues such as medical check-ups and media	[15] [21] [23] [26] [27] [33] [46] [57] [65] [72] [77] [82]	[1]	[19]	[27] [52] [55] [64]
Attentional and interpretation bias to threat-relevant stimuli	[6] [13]	[13] [43]	[6] [71]	**—**
Cognitive vulnerability: intolerance to uncertainty	[6] [41] [65] [71] [79]	[27]	[41]	**—**
Unmet information/knowledge needs	[32]	**—**	**—**	[10] [26] [52] [64] [80]
Social constraints	[11] [69] [81]	**—**	**—**	**—**
Poor problem-solving skills	[31]	**—**	**—**	**—**
Concerns about financial consequences of treatment	[62]	**—**	**—**	**—**
Decision regrets with treatment	[32]	**—**	**—**	**—**
General health worries	**—**	[17] [18] [62]	**—**	**—**
Possibility of potentially negative but uncertain future events	**—**	**—**	[17]	**—**
Inability to interpret and manage treatment-related side effects	**—**	**—**	**—**	[27] [55] [68] [80]
Not being able to rely or count on someone or something	**—**	**—**	**—**	[29]
Complexity, unpredictability, ambiguity of illness	**—**	**—**	**—**	[9] [10] [25] [26] [27] [29] [41] [55] [56] [64] [68] [75] [80]
**Correlates** **=** **23**	**FCR**	**HA**	**Worry**	**Uncertainty**
Younger age	[4] [8] [20] [30] [33] [37] [40] [42] [67] [78] [82]	[38] [40] [62]	[61]	**—**
Excessive emotional distress	[1] [3] [14] [31] [35] [43] [57] [62] [70] [78] [79]	[13] [38]	**—**	[10] [27] [29] [52] [56] [64] [80]
Amount of social support	[56] [69] [81]	[27] [38]	**—**	[55] [64] [80]
Appropriate self-protective response	[21] [39] [66] [73] [76]	**—**	**—**	[39]
Fear of death	[1] [5] [65]	**—**	**—**	[55] [56] [64]
Maladaptive hypervigilant coping	[3] [32] [35] [41] [43] [58] [73] [75]	**—**	**—**	[80]
Difficulties making plans for the future	[3] [15] [65] [70]	**—**	**—**	[29] [80]
Diminished health related quality of life	[2] [3] [11] [21] [22] [23] [33] [37] [43] [57] [60] [65] [67] [70]	**—**	**—**	[27] [64]
Threat appraisal	[50] [60] [66]	**—**	[61]	**—**
Functional impairments	[3] [7] [11] [21] [22] [37] [57] [58] [65] [67] [72] [77] [78] [79]	[58] [59]	**—**	**—**
Specific type of treatment	[42] [47] [67] [76]	[36]	**—**	**—**
Dysfunctional processing of fear	[57]	**—**	**—**	**—**
Chronic uncertainty	[25] [36] [39] [41] [64]	**—**	**—**	**—**
Level of self-efficacy	[82]	**—**	**—**	**—**
Cultural practices	[33] [34] [53]	**—**	**—**	**—**
Depressive symptoms	**—**	[8] [12] [58]	**—**	**—**
Associated with symptom severity	**—**	[8]	**—**	**—**
Associated with self-blame and shame	**—**	[27]	**—**	**—**
Meta-cognitive beliefs about worry	**—**	**—**	[6] [41] [54] [71]	**—**
Level of confidence	**—**	**—**	**—**	[55]
Level of anxiety	**—**	**—**	**—**	[9] [64]
Ability to register information	**—**	**—**	**—**	[25]
Short survival time	**—**	**—**	**—**	[10] [68] [80]

a *Refer to Supplementary File 2 for a complete listing of the references*.

**Table 4:** Measurements: number and prevalence of thematically derived critical attributes for the four constructs: article numbers reflected in Supplementary Material.

**Critical Attribute: measurements**	**Constructs^a^**			
	**Fear of Cancer Recurrence FCR) *n* = 73**	**Health Anxiety (HA) *n* = 38**	**Worry (W) *n* = 11**	**Uncertainty (U) *n* = 15**
Focus group/semi-structured interview with open-ended questions	[1] [16] [21] [27] [39] [65] [73] [75] [77]	[1] [27]	**—**	[27] [29] [39] [55] [68]
Cancer Worry Scale (CWS); Custers et al. (2014, 2018)	[13] [14] [15] [46] [49] [50] [76]	**—**	[61]	**—**
Assessment of Survivor Concerns (ASC) questionnaire; Gotay and Muraoka (1998)	[23]	**—**	[17] [23]	**—**
IES-cancer (measures cancer-specific distress: (a) Intrusive thoughts, and (b) Avoidance; Horowitz et al. (1979)	**—**	**—**	[63]	**—**
Study made 1 item “I worry about my cancer coming back or spreading” from 0 (not at all) to 4 (very much)	[3] [7] [62] [79]	**—**	**—**	**—**
Worry about cancer scale; Easterling and Leventhal (1989)	[2] [12]	**—**	**—**	**—**
Concerns about Recurrence Scale (CARS) (4 domains: worries with health, womanhood, role, and death; Vickberg (2003)	[5] [9] [11] [18] [25] [31] [40] [43] [45] [53] [57] [58] [59] [67] [69] [70] [78] [82]	**—**	**—**	**—**
Fear of Cancer Recurrence Inventory (FCRI) or/and	[41] [42] [67] [71] [72] [73] [74] [76]	**—**	**—**	**—**
FCRI- Subscales or Short Form (FCRI-SF); Simard and Savard (2009)	[6] [14] [16] [35] [44] [58] [60] [63] [69]			
Fear of recurrence questionnaire; Northouse (1981)	[8] [24] [56] [67]	**—**	**—**	**—**
Concerns about Recurrence Questionnaire (CARQ-4); Thewes et al. (2015)	[20] [74]	**—**	**—**	**—**
Visual analog scale, indicating the severity of FCR	[22] [49]	**—**	**—**	**—**
Fears of cancer recurrence scale (FCR7) and short form (FCR4); Humphris et al. (2018)	[30]	**—**	**—**	**—**
Short form of the fear of progression questionnaire (FoP-Q-SF); Mehnert et al. (2006)	[37] [51] [66]	**—**	**—**	**—**
Cancer Rehabilitation Evaluation Survey—Short Form (CARES-SF); Schag et al. (1991)	[47]	**—**	**—**	**—**
FCR-1; Rudy et al. (2020)	[63]	**—**	**—**	**—**
Study made 3-items means (worry about cancer coming back in the same breast, in the other breast, and to other parts of my body) on a 5-point likert-type scale	[32] [33] [34]	**—**	**—**	**—**
Study made items survey yes/no with FCR and fear of death; Befort and Klemp (2011)	[4]			
Study made 5-items FCR empirically derived; Xu et al. (2019)	[81]			
Depression anxiety stress scale-21; Lovibond and Lovibond (1995)	**—**	[6]	**—**	**—**
Hospital Anxiety and Depression Scale (HADS); Zigmond and Snaith (1983)	**—**	[8] [12] [13] [14] [23] [30] [31] [35] [36] [54] [59] [60] [67] [69]	**—**	**—**
State-Trait Anxiety Inventory (STAI); Spielberger et al. (1983)	**—**	[9] [24] [40] [44] [48] [54] [62] [79] [82]	**—**	**—**
Profile of Mood States (POMS) tension–anxiety subscale, by McNair et al. (1971)	**—**	[17]	**—**	**—**
Psychological General Well-being Index (PGWB); Dupuy (1984)	**—**	[18]	**—**	**—**
Schedule for Affective Disorders and Schizophrenia (SADS); Spitzer and Endicott (1975)	**—**	[27] [28]	**—**	**—**
Generalized anxiety disorder scale; Spitzer et al. (2006)	**—**	[35]	**—**	**—**
Numeric visual analog scale for anxiety; Johnson et al. (2016)	**—**	[36]	**—**	**—**
Beck Anxiety Inventory (BAI); Beck et al. (1988)	**—**	[38]	**—**	**—**
Health Anxiety Questionnaire (HAQ); Lucock and Morley (1996)	**—**	[41]	**—**	**—**
Short Health Anxiety Inventory (SHAI); Salkovskis et al. (2002)	**—**	[46]	**—**	**—**
The Breast Cancer Anxiety Scale (BCAS); Kash (2001)	**—**	[48] [62]	**—**	**—**
Profile of Mood States-short form (POMS-SF); Shacham (1983)	**—**	[52]	**—**	**—**
Whiteley Index-Short Form (WI-7); Conradt et al. (2006)	**—**	[74]	**—**	**—**
Metacognitions Questionnaire-30; Wells and Cartwright-Hatton (2004)	**—**	**—**	[6] [71]	**—**
Why do people worry about health questionnaire; Pelletier et al. (2002)	**—**	**—**	[41]	**—**
Penn State Worry Questionnaire (PSWQ); Meyer et al. (1990)	**—**	**—**	[54] [58]	**—**
Illness Worry Scale (IWS); Robbins and Kirmayer (1996)	**—**	**—**	[67]	**—**
Study made 4 items worry about cancer; Easterling and Leventhal (1989)			[19]	
Uncertainty in illness scale-survivor version; Mishel (1999)	**—**	**—**	**—**	[9] [10] [25] [26] [41] [63] [64] [80]
Cognitive Coping Strategies Questionnaire (CSQ); Rosenstiel and Keefe (1983)	**—**	**—**	**—**	[52]
Telephone survey assessing uncertainty triggers; Gill et al. (2004)				[28]

a *Refer to Supplementary File 2 for a complete listing of the references*.

### Stage VI: Consultation Exercise

In this optional step, stakeholders outside the study review team are invited to provide their insights to inform and validate the scoping review findings. We asked members of the IPOS Fear of Cancer Recurrence Special Interest Group (FORwards) for feedback via a short survey. Three individuals provided a critical review, and their feedback was incorporated into our results and discussion.

## Results

### Study Selection and Characteristics

As shown in the PRISMA-ScR flow diagram (see Figure 1), our search strategies initially yielded a total of 3,572 records (2,452 from Medline, 963 from CINAHL, 125 from PsyInfo, and 32 from reference and hand searches). Of these, 3,299 were screened by title and abstract after duplicates were removed. After excluding irrelevant records (*n* = 2,951), 348 full-text articles were retrieved and assessed for eligibility. At this stage, 266 records were excluded. The reasons for exclusion are as follows: irrelevant content (*n* = 168), wrong study population (*n* = 38), English full-text unavailable (*n* = 28), wrong study design (*n* = 25), and duplication (*n* = 7). A final set of 82 articles met all inclusion criteria and were included in this review (refer to Supplementary File 2 for a complete listing of the references).

Extracted data (see Table 5) included the description of studies by location, sample size, and study design. The majority of studies used a quantitative, cross-sectional survey design (50.0%, *n* = 41) that had 101–500 participants (*n* = 39). Nearly 48% of the included studies were conducted in the United States (*n* = 39).

**Table 5:** Description of studies by location, sample size, and study design (*N* = 82): article numbers reflected in Supplementary Material.

	**Article Number^a^**	***n***	**(%)**
**Location by Country**			
Australia	[6] [18] [71] [72] [73] [74]	6	7.3
New Zealand	[12]	1	1.2
Netherlands	[13] [14] [15] [16] [76]	5	6.1
Demark	[20]	1	1.2
German	[37] [51]	2	2.4
Turkey	[38] [65]	2	2.4
France	[44] [54]	2	2.4
United Kingdom	[1] [2] [28] [30] [75]	5	6.1
Thailand	[5] [8] [80]	3	3.7
Tai Wan	[21] [22] [23]	3	3.7
Japan	[31] [53] [57] [59]	4	4.9
China	[60] [81]	2	2.4
Korea	[66]	1	1.2
Canada	[40] [41] [42] [63] [67] [68]	6	7.3
United States	[3] [4] [7] [9] [10] [11] [17] [19] [24] [25] [26] [27] [29] [32] [33] [34] [35] [36] [39] [43] [45] [46] [47] [48] [49] [50] [52] [55] [56] [58] [61] [62] [64] [69] [70] [77] [78] [79] [82]	39	47.6
**Sample size**			
1–50	[1] [9] [16] [21] [29] [31] [36] [39] [46] [54] [55] [56] [65] [68] [70] [73] [75] [76] [77]	19	23.2
51–100	[2] [5] [6] [7] [35] [43] [44] [59] [63] [64] [81]	11	13.4
101–500	[3] [8] [10] [11] [12] [13] [15] [17] [18] [19] [20] [22] [23] [24] [25] [26] [27] [28] [30] [38] [41] [42] [45] [48] [49] [50] [53] [57] [58] [60] [61] [62] [66] [69] [71] [72] [78] [79] [80]	39	47.6
501–1,000	[4] [32] [47] [52] [67]	5	6.1
>1,000	[14] [33] [34] [37] [40] [51] [74] [82]	8	9.8
**Study design**			
Qualitative interviews	[1] [16] [21] [27] [28] [29] [39] [55] [65] [68] [73] [75] [77]	13	15.9
Instrumental validation	[14] [30] [42] [60] [63] [67] [74] [78]	8	9.8
Case study	[46] [54] [76]	3	3.7
Cross-sectional surveys	[4] [6] [7] [8] [9] [10] [11] [12] [13] [15] [18] [19] [20] [22] [24] [33] [34] [36] [37] [38] [40] [41] [44] [45] [48] [50] [51] [53] [56] [57] [58] [60] [62] [64] [66] [70] [71] [72] [80] [81] [82]	41	50.0
Longitudinal or prospective	[32] [47] [49]	3	3.7
Randomized controlled trials	[23] [25] [26] [35] [43] [52] [59]	7	8.5
Mixed method	[2] [69]	2	2.4
Other designs	[3] [5] [17] [31] [79]	5	6.1

a *Refer to Supplementary File 2 for a complete listing of the references*.

### Critical Attributes Part I: Conceptualization

#### Characteristics

**Figure 2:**
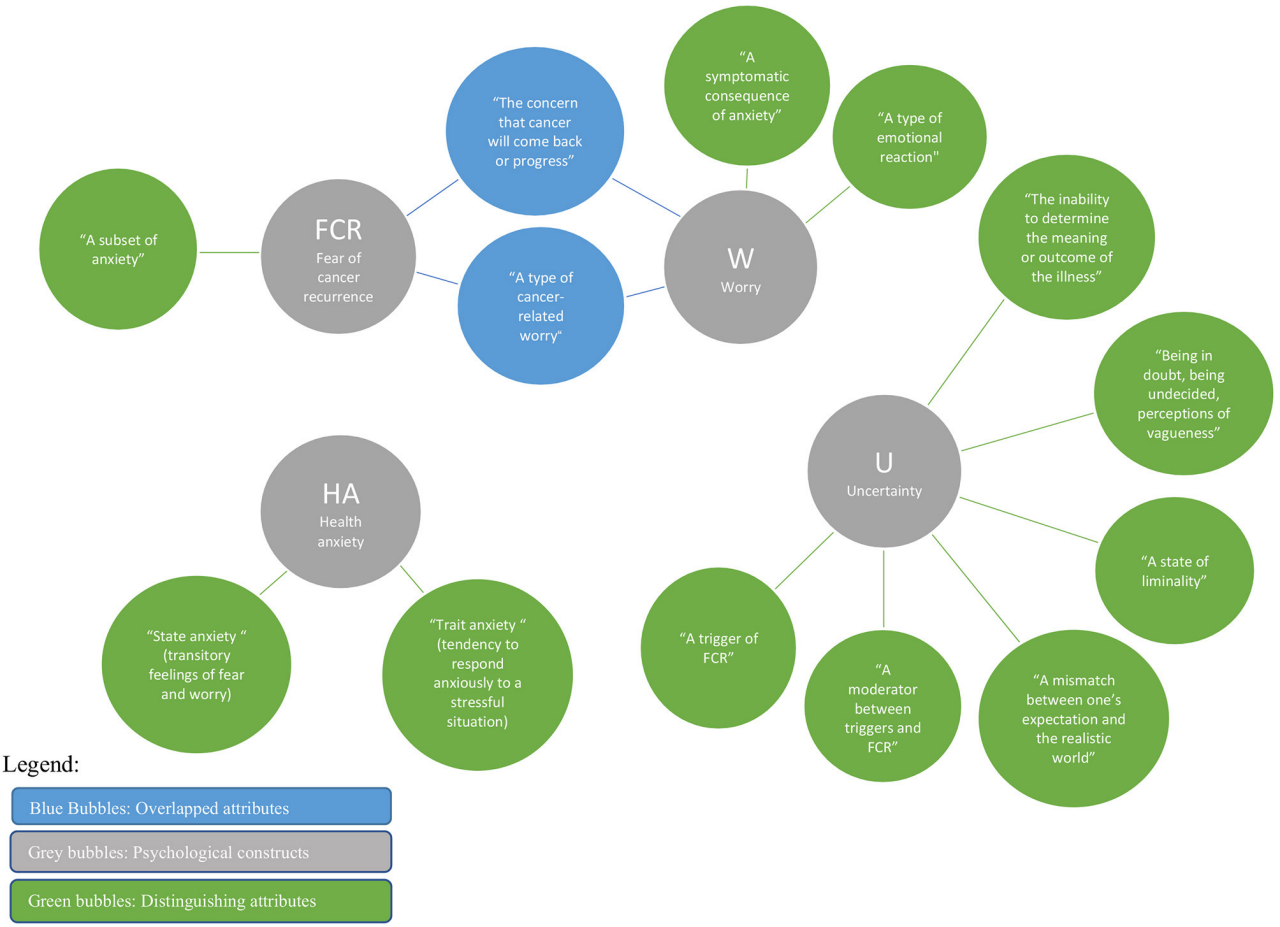
Overlapped and distinguishing characteristics of the four constructs.

As seen in Table 3 and Figure 2, 13 descriptions of characteristics were identified from the four analyzed constructs. While overlaps were found among certain study constructs, not one characteristic was found to be shared among all four constructs. Two characteristics identified with HA alone are *state* and *trait anxiety. Six* characteristics were identified only for uncertainty, which included: *the inability to determine the meaning or outcome of the illness; being in doubt; a state of liminality; a mismatch between one's expectation and the realistic world; a moderator between triggers and FCR;* and *a trigger of FCR*. These unique characteristics speak to the vagueness and liminality of a situation or illness outcome and could include risk of recurrence. One unique characteristic was identified for FCR: *a subset of anxiety;* the two unique descriptions for worry included: *a symptomatic consequence of anxiety* and *a type of emotional reaction*. Two characteristics were shared by both FCR and worry about breast cancer: *the concern that cancer will come back or progress*, and *a type of cancer-related worry*.

#### Theoretical Features

**Figure 3:**
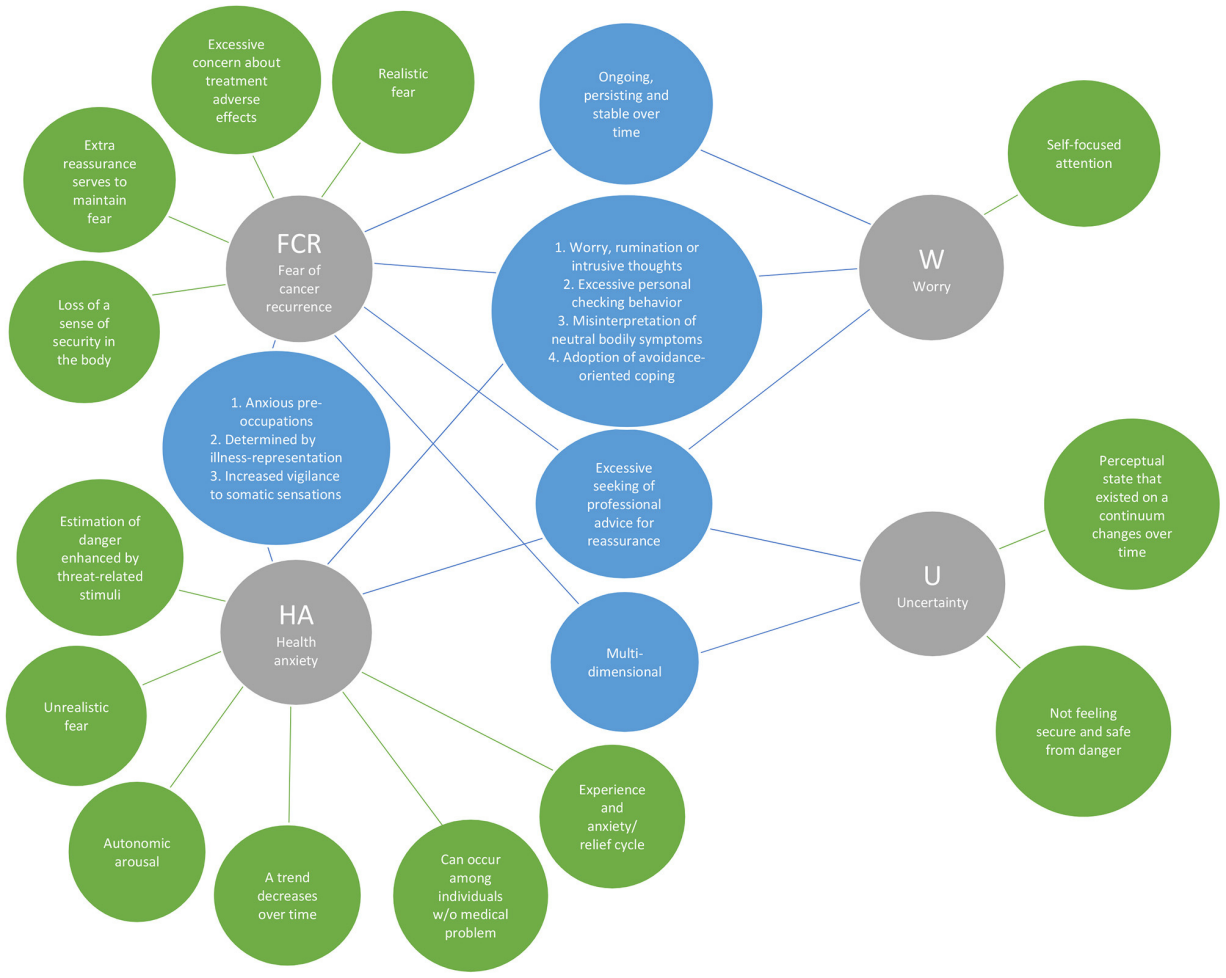
Overlapped and distinguishing theoretical features of the four constructs.

As seen in Table 3 and Figure 3, 23 theoretical features were identified, 10 of which were observed in more than one construct. One feature, *excessive seeking of professional advice for reassurance*, was identified in all four constructs. FCR, HA, and worry had four additional theoretical features in common, they all involved *excessive personal checking behavior; misinterpretation of neutral bodily symptoms; adoption of avoidance-oriented coping;* and *worry, rumination or intrusive thoughts. Three* other shared features were found for FCR and HA: *being determined by illness representation; increased vigilance to somatic sensations;* and *anxious preoccupations*. In addition to the above-mentioned shared features, FCR and worry shared one unique feature: o*ngoing, persisting, and being stable overtime*. *Multidimensionality* was a theoretical feature observed in both FCR and uncertainty, along with the main common features shared in all four constructs with *excessive seeking of professional advice for reassurance*.

Distinguishable theoretical features of each of the four psychological constructs were identified. Studies examining FCR recognized four unique theoretical features of this construct: *realistic fear; excessive concern about the treatment adverse effects; extra reassurance serves to maintain patients' fear;* and *loss of sense of security in the body*. Those investigating HA identified four distinct features: the *estimation of danger is enhanced by threat-related stimuli; unrealistic fear; autonomic arousal (apprehension, tension and nervousness); decreases over time; can occur among individuals without a medical problem*, and sometimes involves an *anxiety/relief cycle. Self-focused attention* was the single unique theoretical feature identified in the literature with worry. Unique theoretical features of uncertainty include a *perceptual state that exists on a continuum changing over time* and *not feeling secure and safe from danger*.

#### Triggers

**Figure 4:**
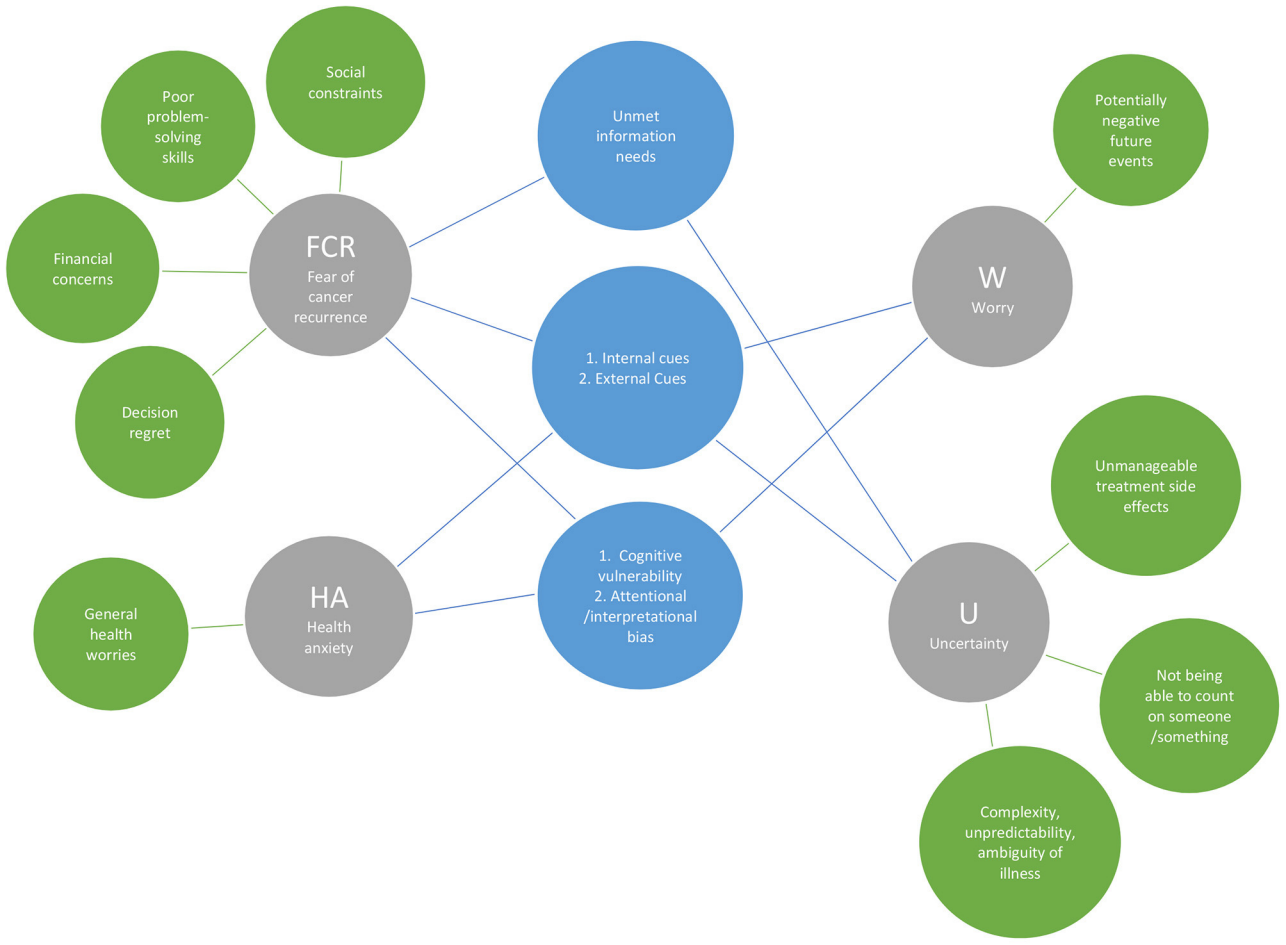
Overlapped and distinguishing triggers of the four constructs.

A total of 14 triggers were identified from the four constructs as seen in Table 3 and Figure 4. Five of these were observed in more than one construct. FCR, HA, worry, and uncertainty had two triggers in common; they are all triggered by *internal cues* (e.g., somatic/physical symptoms) and *external cues* (e.g., medical check-ups and media). For all constructs except for uncertainty, two common triggers were found such as *cognitive vulnerability (i.e., Intolerance to uncertainty*); and *attentional and interpretation bias to threat-relevant stimuli*. Both FCR and uncertainty were triggered by *unmet information/knowledge needs*.

Several distinguishing triggers of each construct were also identified. The unique triggers of FCR were *social constraints; poor problem-solving skills; concerns about financial consequences of treatment;* and *having regret about treatment decisions*. Uncertainty was specifically triggered by *unmanageable treatment side effects*; *not being able to rely on count on someone or something;* and *the comple*x, *unpredictable and ambiguous nature of the illness*. A unique trigger of HA was *general health worries*, and the *potentially negative but uncertain future events* was a distinctive triggering factor for worry.

#### Correlates

**Figure 5:**
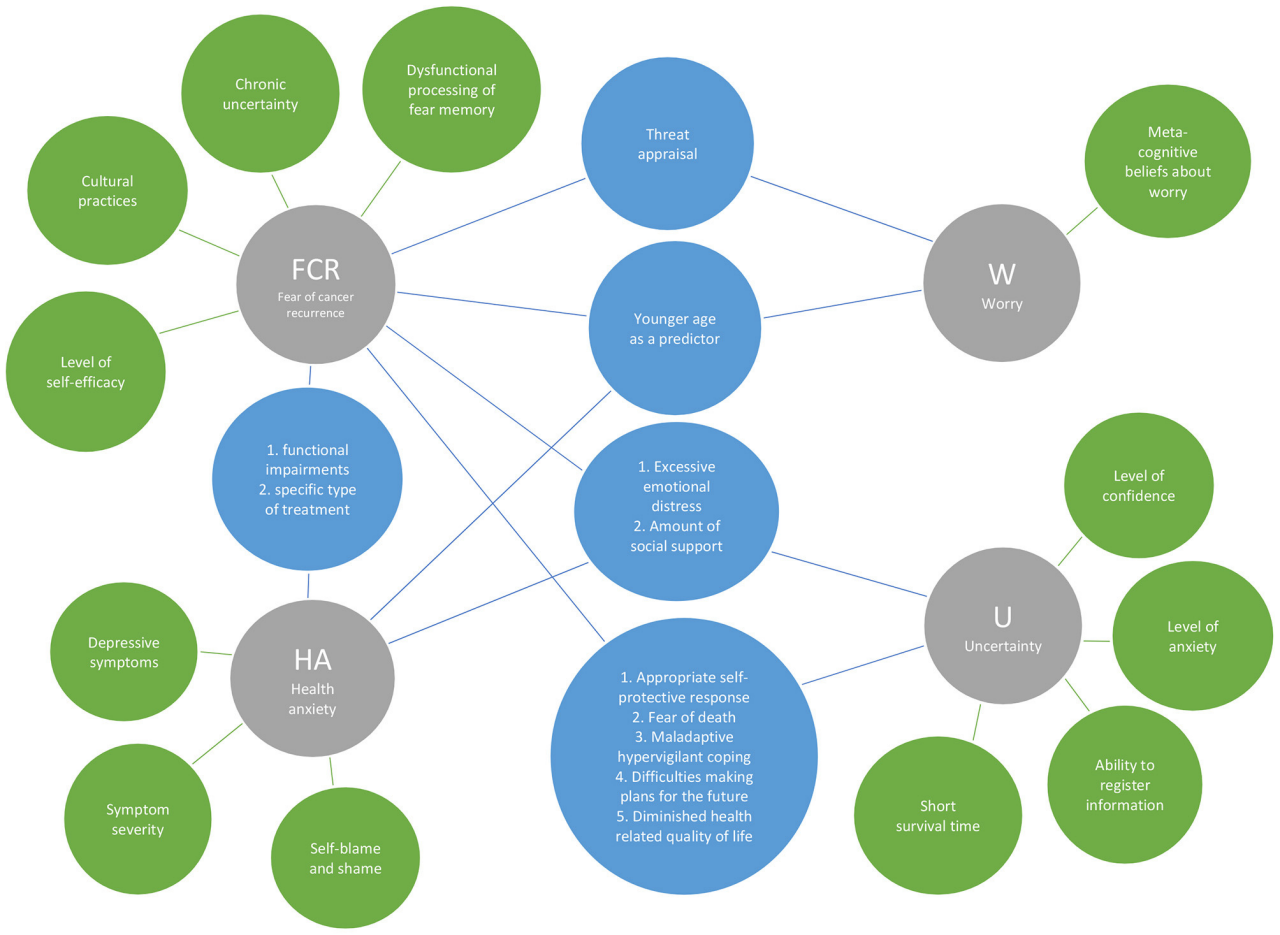
Overlapped and distinguishing correlates of the four constructs.

As outlined in Table 3 and Figure 5, among the 23 correlates gathered from the 82 selected papers, 15 were associated with FCR, 8 with HA, three with worry, and 11 with uncertainty. No correlates were identified with all four constructs. At most, three correlates were identified with three of the constructs: *excessive emotional distress* and *amount of social support* with FCR, HA, and uncertainty; and *younger age* with FCR, HA, and worry. Nine correlates were identified with various combinations of two constructs with FCR being reflected in all of these combinations.

Twelve correlates were identified with only one of the study constructs. Correlated only with FCR were: *dysfunctional processing of fear memory; chronic uncertainty; level of self-efficacy;* and *cultural practices*. Correlates identified with HA were *depressive symptoms, symptom severity*, and *self-blame and shame*, and worry was the only construct identified with *metacognitive beliefs about worry*. Lastly, uncertainty was the only construct linked to *level of confidence, level of anxiety, ability to register information*, and *shorter survival time*.

### Critical Attributes Part II: Measurements

As presented in Tables 4, 6, the construct of FCR was measured using 18 different approaches from the 73 FCR papers retained. The three most frequently used scales to measure FCR were the Concerns About Recurrence Scale (CARS) (Vickberg, 2003) used 18 times, followed by the Fear of Cancer Recurrence Inventory (FCRI) (Simard and Savard, 2009) used 17 times (8/17 times full form, and 9/17 times short forms severity subscale), and the Cancer Worry Scale (CWS) used seven times with either the 8-items (Custers et al., 2014) or the revised 6-items scale (Custers et al., 2018). Two scales were used to measure either FCR and worry: the CWS (Custers et al., 2014), and the Assessment of Survivor Concerns (ASC) scale (Gotay and Pagano, 2007). In examining the development of these scales, the CWS by Custers et al. (2014) is the only scale that was developed to specifically measure FCR, but not worry, in breast cancer survivors and contains subscales (e.g., cognition, intrusiveness, and general worry with cancer) that can also be found in the other two scales mentioned above. CWS is the only scale among the three to have a specific cut-off score that distinguishes between low and high FCR levels. As for the ASC scale, it is not specific to fear of cancer recurrence and includes broad assessments of HA. While FCR and worry shared similar tools to measure two different constructs, worry was measured using seven different approaches and uncertainty measured using four different approaches (see Table 4). HA and uncertainty did not have overlapping measures to other study constructs. Among the 38 HA studies, the most consistently cited scale to measure this construct was the Hospital Anxiety and Depression Scale (HADS) (Zigmond and Snaith, 1983) with 13 mentions. Among the measures used for worry, only the PSWQ represents a trait measure (Meyer et al., 1990). Uncertainty was measured eight times using the Mishel Uncertainty in Illness scale (MUIIS) (Mishel, 1981).

**Table 6:** Number and frequency of use of FCR, U, HA, and W Measurements.

**Constructs and number of measurements^a^**	**Measurement tools and number of times used/study # in decreasing number^b^**
73 articles examining **FCR**, 15 measurement approaches were used	**CARS** #18; **FCRI** #17; **Semi-structured interviews;** #9; **CWS** #7; **FRQ** #4; **1 item Worry about cancer coming back** #4; **FoP-Q-SF** #3; **3 group items** #3; **CARQ-4** #2; **Worry about Cancer Scale** #2; **Visual Analog Scale** #2; **FCR7/FCR4** #1; **ASC** #1; **FCR-1** #1; **CARES-SF** #1
38 articles examining **HA**, 15 measurement approaches were used	**HADS** #13; **STAI** # 9; **BCAS** #2; **SADS** #2; **Semi-structured interviews** #2; **POMS** #1; **POMS-SF** #1; **PGWB** #1; **DASS-21** #1; **GAD-7** #1; **NVAAS** #1; **BAI** #1; **HAQ** #1; **SHAI** #1; **WI-7** #1
11 articles examining **Worry**, 7 measurement approaches were used	**ASC** #2; **Metacognitions Questionnaire** 30 #2; **CWS** #1; **IES-cancer** #1; **WWQ** #1; **PSWQ** #2; **IWS** #1
15 articles examining **Uncertainty**, 3 measurement approaches were used	**MUIIS** #8; **Semi-structured interviews** #5; **CSQ** #1; **Telephone survey** #1

a *It is possible that more than one construct is examined in a single study*.

b *Refer to Table 4, for abbreviations of the measurement tools*.

## Discussion

This scoping review represents the first known study that has used a rigorous knowledge synthesis methodology to simultaneously examine four similarly viewed psychological constructs in the breast cancer survivorship literature: FCR, HA, worry and uncertainty in illness. This review identified the unique and overlapping conceptual components of these constructs and examined the instruments used to assess them. Findings reveal a partial overlap among the four constructs with some uniqueness in their critical attributes (i.e., characteristics, theoretical features, triggers and correlates), but less overlap in their measurement.

### Critical Attributes: Conceptualization (Characteristics, Theoretical Features, Triggers, Correlates) and Measurement

This review found that the four study constructs share many attributes, which may explain in part the initial conundrum which was the catalyst for conducting this review: their frequently interchangeable use in the empirical literature (Bradford et al., 2013; Jones et al., 2014; Butow et al., 2019). In this section, we aim to bring clarity on their categorical distinctions and areas of overlap.

### Overview of Unique Attributes

The principal finding of our scoping review was that the psycho-oncology literature on breast cancer yields significant attributes that delineate clear distinctions among the four constructs of interest: FCR, HA, uncertainty, and worry. Beginning with the analysis of characteristics (see Table 3 and Figures 2–5), among the 13 characteristics (see Figure 2) identified in the 82 papers reviewed, none of the characteristics overlapped all four constructs, 11 were distinguishing characteristics, and only two were shared by two constructs (FCR and worry). Similarly, 13 (of 23) theoretical features, nine (of 14) triggers, and 12 (of 23) correlate attributes were specific to only one construct. Thus, these findings from across the four critical attributes used in our analysis—characteristics, theoretical features, triggers, and correlates—provide a fruitful point of departure for delineating key distinctions among FCR, HA, worry and uncertainty.

Characteristics attributes that were unique to FCR include *the concern that cancer will come back or progress, a type of cancer-related worry, decision regrets with treatment, concerns with financial consequences of treatment, hypervigilance coping, memory dysfunction, poor self-efficacy and cultural practices, difficulty in making plans for the future*. Some of these identified FCR characteristics resonate with those identified from an international Delphi survey identifying key characteristics of FCR (Mutsaers et al., 2019), with some distinctions found. That is, other FCR characteristics identified in this scoping review are *decision regrets with treatment, concerns with financial consequences of treatment, and cultural practices*.

Another unique trigger related to FCR and not associated with the other three constructs was for social constraints, financial worries, and treatment decision regrets (Janz et al., 2014). The findings of our review and the recent Delphi survey support the notion that FCR may likely be best assessed as a multidimensional concept (Mutsaers et al., 2019). FCR also tended to remain unchanged if not clinically addressed whereas the other constructs were observed to carry elements of change over time. In our findings, FCR was the only construct aligned with the attribute that “*extra reassurances serve to maintain the fear*.” Another subtle difference found between FCR and the other three constructs was the increased frequency of threat appraisal associated with FCR. This finding may reflect the literature focused on providing for exposure therapy with a specific focus on fear. In contrast, the other constructs who remain more general tend to be treated with behavioral therapy. This would be a topic to study more widely.

The correlate cultural practices was only identified with FCR (Janz et al., 2011, 2016; Momino et al., 2014). Janz et al. (J2011) and Janz et al. (2016) found higher worry of recurrence (as they named) among Latina women in comparison to White women, while African American women experienced lower worry of recurrence than White women. The authors suggested that the main factors for higher worry of recurrence among Latinas were attributed to having less information about cancer, longer delays in diagnosis, and poorer communication styles. These findings might be due to the different levels of cultural appropriateness of expressing FCR among cultures, or the availability of psychosocial oncology assessment and/or intervention in different geographic locations. However, it should be noted that an examination of FCR correlates across cultures or countries has received little empirical study. This gap illuminates an important area for expanded research, as FCR measures are translated into an increasing number of languages [e.g., Chinese (Lin et al., 2018), Dutch (van Helmondt et al., 2017), and Persian (Bateni et al., 2019), to name a few]. Scholars such as Momino et al. (2014) have illuminated key cultural differences when validating the American-developed Concerns About Recurrence Scale (Vickberg, 2003) among Japanese participants. Slight differences in the factor structure were found between American and Japanese women; in the Japanese version, they found a new factor reflecting continuity after death widely believed to exist in this country. Collectively, these findings suggest that cultural differences impact the degree to which FCR, and perhaps other psychological problems, are experienced or displayed.

HA presented itself with six unique theoretical features that could point to a definition of HA itself (i.e., *estimation of danger, unrealistic fear, autonomic arousal, decreases over time accompanied by anxiety and relief cycle, and can be present in individuals with no known medical problem*). Even the relief cycle can also be helpful in differentiating between HA and generalized anxiety disorders, as the latter may be psychopathological conditions for which relief is unlikely to be shown (Dugas et al., 1998). However, in this review, HA was most often defined using terms such as a transient feeling to respond anxiously with fear or worry to a potential or existing health threat. Using HA as a proxy for FCR only becomes relevant if the worried thought and emotion is anchored to a context associated with FCR such as feeling of fear or worry that cancer will come back. Otherwise, HA represents a general state of worry toward one's health. Interestingly, HA was the only construct correlated with self-blame and shame and depression (Gill et al., 2004) and had distinguishing characteristics of both “*trait anxiety*” and “*state anxiety*.” Previous evidence has revealed the correlation between shame proneness with maladjustment (Tangney et al., 1992) and the correlation between self-blame and major depressive disorder (Zahn et al., 2015). More specifically, it was argued that shame and guilt were similar in terms of internal attributions, but they also differ since shame had global and stable attributions while guilt had specific and unstable attributions for negative events (Tangney et al., 1992). Therefore, given these findings, it can be suggested that HA, unlike other constructs, may show characteristics of both transient distress and psychopathology depending on the level of effect on self-concept. In contrast, a recent review on the correlates of FCR found that this concept was on the whole rather stable, or perhaps could initially decrease then stabilize (Lebel et al., 2020). Worry was uniquely related to the theoretical attribute of self-focused attention. This finding can be particularly important when designing a measurement or also an intervention program, as individuals who are worriers tend to worry about themselves in specific situations, while in FCR, the situations feared are more varied (*feeling nervous prior to doctor's appointments, worrying about what will become of the family, being afraid of becoming less productive at work, fear of leaving the children parentless, fear of further treatment, fear of dying*) (Götze et al., 2019).

Uncertainty was the only construct where its defining characteristics were existential issues aligned with the concept of uncertainty (i.e., *being unable to determine meaning of illness outcome, being doubtful, mismatch between own expectations and real world*). However, uncertainty and FCR did share overlapping triggers related to patient information needs that could be considered in designing preventative programs by providing good and sound information tailored to patient needs.

### Overlapping Attributes

Among the identified theoretical features, several substantial features overlapped among the four constructs, with a majority of features being behavioral cues. For example, the increased use of and seeking professional advice for reassurance along with excessive personal checking behavior was often noted as a consequence of FCR (Janz et al., 2011, 2016); these behavioral cues, however, were found to be associated with all four constructs. Therefore, these attributes are not strong delineating characteristics.

*Internal cues* (e.g., somatic/physical symptoms) and *external cues* (e.g., medical check-ups and media) are identified as triggers for all four constructs. This finding was expected, perhaps related to seeking reassurance, since both internal and external cues are somatic related (Hall et al., 2017). External triggers are also related to media exposure and could be the stimuli for non-cancer specific psychological disturbance such as HA and worry, as well as cancer-specific distress including FCR and uncertainty of illness (Lemal and Van den Bulck, 2009). Furthermore, FCR, HA and uncertainty shared two overlapping triggers. First, the trigger *cognitive vulnerability to uncertainty* overlapped with the three constructs, but interestingly, not with uncertainty. This finding was perhaps due to the nature of the analytical methodology, as studies addressing the construct of uncertainty would not necessarily discuss “*cognitive vulnerability to uncertainty*” as a trigger. An alternative explanation could be that this trigger is a better fit to intolerance of uncertainty, a trait like characteristic associated with the tendency to react negatively, emotionally, cognitively, and behaviorally, to uncertain situations (Buhr and Dugas, 2006). The trigger *attentional/interpretational bias* was found to overlap between HA and worry but not FCR. These results are consistent with previous findings linking attentional bias with HA and worry (Butow et al., 2015; Custers et al., 2015; Ng et al., 2020) (not FCR) whereby anxious people are found to pay increased attention to threatening stimuli (attentional bias) (Lees et al., 2005; Kaur et al., 2013; Thewes et al., 2013; Aue and Okon-Singer, 2015). Finally, one trigger, *unmet information needs*, was identified in both FCR and uncertainty and is consistent with the literature. Insufficient and inaccurate information about cancer prognosis and recurrence risks are known to contribute to fear and uncertainty among breast cancer survivors (Lebel et al., 2014, 2018, 2020).

Within the correlates tabulated in Table 3, *younger age* was associated with FCR, HA, and worry, but not uncertainty. The finding that younger cancer patients are more likely to experience FCR, HA, and worry is supported in the literature (Janz et al., 2011; Jones et al., 2014; Mirosevic et al., 2019; Chumdaeng et al., 2020), and the reasons may be related to their responsibilities at this time in their life. For instance, younger cancer survivors are more likely to have dependent children and thus their FCR might relate to the future care of their children if their cancer should recur (Maheu, 2009). Similarly, younger cancer survivors are more likely to be engaged in employment (Stone et al., 2017) and therefore may be concerned with work-related problems. However, the lack of association between younger age and uncertainty does not necessarily mean that an association does not exist; on the contrary, younger persons with cancer do experience cancer-related uncertainty which can lead to negative effects (Wonghongkul et al., 2000; Corbeil et al., 2009; Kim et al., 2012). Therefore, the lack of association between younger age and uncertainty found in this review may be due to the lack of literature examining this association, rather than the true absence of it.

Excessive emotional distress and amount of social support were associated with FCR, HA and uncertainty, but not with worry. Notably, both FCR and HA were also associated with *functional impairments* (i.e., *physical, emotional, and social impairments*), which add contextual considerations for how women with breast cancer may make sense of their diagnosis. Indeed, context is theorized to play an important role in such processes: Leventhal's Common Sense Model (Leventhal et al., 2016) posits that the socio-cultural context (e.g., *amount of social support*) as important to interpret illness (e.g., *functional impairments*) and emotional (e.g., *excessive emotional distress*) outcomes. The Common Sense Model (Leventhal et al., 2016) would refer to an illness representation as well as increased vigilance in somatic sensations (Freeman-Gibb et al., 2017; Richardson et al., 2017; Tsai et al., 2018; Petricone-Westwood et al., 2019). Implications for researchers or clinicians could be the need for interventions tailored to specific treatment types; in other words, breast cancer patients receiving these specific treatments might experience higher levels of impairment that warrant particular interventions to also target their fear and anxiety. It should include techniques to reduce excessive somatic vigilance in both cases.

FCR and worry share the threat appraisal feeling. This emotion is not only related to the perceived vulnerability of recurrence, but also its grade or severity. Clinically, it is important to consider both aspects, the concept and its intensity as related to worry. Both constructs were identified in this review as ongoing, persistent, and stable over time. Given this potential for FCR and worry to persist as long-lasting conditions if left unaddressed, their interventions must be incorporated into follow-up cancer survivorship plans.

Related to FCR, HA and uncertainty, they are assessed as multi-dimensional concepts (McCormick, 2002; Simard et al., 2010) and as a strength, they are all related to an *appropriate self-protective response*. However, conversely, they represent a hypervigilant, maladaptive way of coping that may interfere with the ability to make future plans, highly related in both cases and identified in this review with attributes such as “*fear of death*”; and “*diminished health related quality of life*.”

### Attributes That Could Help Distinguish Between Two or More Constructs

Third, our review identified several attributes that could potentially be used to draw boundaries between the four constructs, but which warrant further investigation. In particular, the level and duration or severity of each construct is instrumental in its diagnosis and treatment. Here we draw on cognitive-behavioral and common-sense theoretical models to expand upon our findings.

The cognitive-behavioral model of HA (Salkovskis and Warwick, 2001) is the most prominent theoretical framework. In the cognitive-behavioral model, dysfunctional beliefs about bodily sensations (e.g., “being healthy means being free from bodily sensations”; “if there are unpleasant bodily symptoms, it must be a sign of serious illness”) and negative images (e.g., imagining oneself as having a fatal disease) lead to physiological arousal and emotional distress. In turn, to cope with this distress, maladaptive safety behaviors lead to a vicious, on-going cycle, for example, of reassurance, help-seeking, and body checking (Salkovskis and Warwick, 2001). Accordingly, in their meta-analysis Marcus et al. (2007) related HA to beliefs that physical sensations are harmful, and illnesses are uncontrollable and inevitable. These beliefs are maintained by selective attention to health threats (Owens et al., 2004) and further exacerbated by safety behaviors (Olatunji et al., 2011).

From the cognitive-behavioral perspective, several overlapping theoretical features found in this review are “*worry, rumination or intrusive thoughts*,” “*misinterpretation of bodily symptoms*,” “*adoption of avoidance-oriented coping*,” and “*excessive personal checking*” behaviors. In addition, the result that both FCR and worry were described as constructs that are *ongoing, persisting, and stable over time* might be considered as common indicators of a higher level of FCR, worry, and HA. Yet symptoms of high levels of FCR can meet the former diagnosis of hypochondriasis in DSM IV (Thewes et al., 2013). More specifically, symptoms of excessive and recurrent intrusive thoughts and somatic sensations were the sign of serious illness of higher level FCR and consistent with the diagnosis criteria of somatic symptom disorders of DSM-5. Indeed, higher levels of persistent FCR are defined by maladaptive/emotion-focused coping strategies (Lebel et al., 2016b). All in all, although FCR is not recognized as a mental illness or a psychiatric condition, high levels of FCR or HA may result in pathological psychological disorders that necessitate professional intervention and/or treatment within the CBT framework.

Other overlapping correlates of “*functional impairments*” and “*a specific type of treatment (i.e., chemotherapy and mastectomy)*” of both HA and FCR might be addressed within the context of the common-sense model. Thus, in the early FCR model of Lee-Jones et al. (1997) in which the common-sense model was utilized, the hypothesis that perceptions regarding the consequences of illness and treatment control were the factors that correlated with FCR was supported by the evidence (Llewellyn et al., 2008; Corter et al., 2013).

### Critical Attributes: Measurement

Upon analyzing the approaches used to measure all of the four studied constructs, we found that the majority were on par with their intended constructs. Eighteen approaches were used to measure FCR, 15 for HA, eight for worry, and four for uncertainty. Examples of scales used to measure only one construct include for FCR, the Concerns About Recurrence Scale (CARS) (Vickberg, 2003) and the Fear of Cancer Recurrence Inventory (Simard and Savard, 2009), whereas, for worry, the Penn State Worry Questionnaire (Meyer et al., 1990) was mostly used. Uncertainty was mainly measured using the Uncertainty in Illness Scale (Mishel, 1981). Nevertheless, as opposed to using instruments specifically developed to measure HA, this construct was found to be measured mostly using non-specific anxiety scales such as the Hospital Anxiety and Depression Scale (Zigmond and Snaith, 1983) and the State-Trait Anxiety Inventory (Spielberger et al., 1983). Among the 38 HA studies, the only HA-specific instrument used to measure this construct was the Health Anxiety Questionnaire (HAQ) (Lucock and Morley, 1996). This observation is unusual; some of the most widely used and psychometrically validated instruments of HA (Hedman et al., 2015), such as the Health Anxiety Inventory (HAI) (Salkovskis et al., 2002) and the Whiteley Index (WI) (Pilowsky, 1967) have not been found in our sample.

The literature shows that there is still ongoing reflection as to whether these psycho-oncology constructs are best conceptualized and measured as multidimensional or as independent constructs with unique features (Costa et al., 2016; Costa, 2017; Galica et al., 2018; Maheu and Galica, 2018). Some proponents will argue that our understanding of the construct such as FCR would be best served as a unique construct, rather than aggregating its associated dimensions into a total FCR score (Costa, 2017; Maheu and Galica, 2018). The results of this scoping review provide guidance to assist in identifying main and distinctive features and underlying dimensions to each of the four studied constructs.

## Recommendation for Practice

The unique features and differences revealed by this review among the four constructs can provide useful guidance for the design of targeted intervention programs. While the anxiety reduction approach would be important for the four constructs, other factors may yield important impacts. For instance, in FCR, perhaps a broader approach that includes objectives of the cancer survivor would be more effective (i.e., advice or help for work, coping with children communication, and financial concerns) due to the presence of these triggers in FCR. A program should also include general information specially focused on FCR and levels of uncertainty. Moreover, in the development of FCR interventions, both existing CBT methods developed for HA, worry, and uncertainty (due to the common antecedents/triggers, maintaining factors, and consequences) and components of the common-sense model should be taken into account in order to provide a comprehensive illness-specific intervention.

The review results illustrate a consistent behavioral component in the excessive search for relief from a professional (more frequent consultations than required caused by fear or suspicion of physical discomfort or pain that could indicate a relapse). This aspect should be taken into account when designing a treatment program since response prevention is an effective tool. This approach also may work to reduce excessive somatic vigilance, more frequent in FCR and HA. However, behavior-focused psychotherapeutic approaches are not required for uncertainty, a cognitive and emotional construct, for which behavioral patterns such as checking bodily symptoms, seeking reassurance, and the avoidance of stimuli are absent. Younger age was identified as a similar feature for FCR, HA and worry. Hence, when screening for individuals at risk of psychological strain due to cancer, we can assume that three of our four constructs could be at play with younger individuals (Petricone-Westwood et al., 2019). Based on the distinctive and unique features of each of the constructs under review, the general recommendations for practice are as follows. When the focus of a study becomes the concern that cancer will come back or progress, one is looking at FCR. When the focus is on assessing the emotional reaction to cancer, then likely the best construct to study would be worry. In lieu of the association between intolerance of uncertainty and worry, when worry leads to negative behavioral reactions, an intolerant prone trait should be considered (Gu et al., 2020). However, when pre-existing psychological states and their impacts on FCR are in the forefront, then health anxiety should be the primary focus. Finally, when the focus sits more on that state of liminality associated with living with an illness that may return such as cancer (Pilowsky, 1967), then the construct at hand is uncertainty related to the illness.

## Recommendation for Research

This review identified the similarities and differences in the four constructs experienced by women living with breast cancer. Our review of the key attributes of each construct studied will guide researchers in future reviews and theory development. The observed overlap implies an incomplete understanding of the etiology of these psychological constructs, which hinders the thoughtful selection of interventions based on a specific understanding of the target construct and underlying mechanisms. In our review, three out of four of the constructs (i.e., FCR, worry, uncertainty) were measured using specifically focused construct measures. This finding suggests that future research into the unique attributes of each construct can be carried out by deconstructing these measures into their unit scales and conducting correlations studies using path analyses. Such research findings would support the delineation of unique and specific attributes for each construct. For HA, which was found to be measured with non-specific anxiety scales, the recommendation would be to use a HA-specific instrument to measure this construct, such as the Health Anxiety Inventory (Salkovskis et al., 2002), a valid diagnostic instrument that will allow researchers to differentiate HA level between patients with medical problem (e.g., cancer survivors) than those without any illness.

## Limitation and Conclusion

We acknowledge several limitations of this review. First, there is a potential omission of relevant evidence due to our exclusive focus on breast cancer survivors' research. Potential interpretation bias might also be present in the classification of the critical attributes, despite the use of duplicate independent review of articles during data extraction and analysis. As with all reviews, this scoping review is limited by the quality of evidence being analyzed. Particularly with regard to findings related to the correlates of the four constructs, the majority of primary studies lacked the longitudinal design required to infer temporal relationships between variables. Our review relies on the conceptual and operational definitions of the authors of the primary studies. Moreover, this review followed a scoping review method, which unlike systematic reviews, do not go through the process of quality assessment. As a result, this review might have left the congruence of measures used unchecked. Further, we reviewed the theoretical features but not the original theoretical frameworks related to each of the constructs. Future studies could analyze how researchers apply different theories and models to guide their research into the four constructs.

Despite these limitations, this scoping review established key distinguishing critical attributes of the four psychological constructs, which is necessary for improving consistency in identification criteria/conceptualization and measurement. Our findings have the potential to inform more rigorous approaches to investigating the psychological impact of breast cancer diagnosis and treatment on survivors.

More work is needed to conceptually differentiate these constructs, as our findings indicate that attributes across constructs overlap. For instance, to move forward with the conceptualization of FCR, it is essential for researchers to choose differentiating languages to describe each of the studied constructs and perhaps contrast them against FCR to identify differences. We recommend using the unique critical attributes that were found in this review, highlighted in Table 3 to assist in this differentiation. For HA, our findings indicate that its attributes (e.g., its triggers) were very similar to that of anxiety in general, which is not necessarily triggered by cancer diagnosis. Worry was the least mentioned construct according to our search, and it appeared to be more cognitively associated, and was described as a consequence of anxiety triggered by somatic or other external cues. Although not conclusive, our findings offer valuable insights into the unique features that assist researchers and clinicians to differentiate the four psychological constructs, which will eventually contribute to more tailored and targeted care for breast cancer survivors.

## Data Availability Statement

The original contributions presented in the study are included in the article/Supplementary Material, further inquiries can be directed to the corresponding author/s.

## Author Contributions

CM and MS: study design and conceptualization. CM and WLT: project coordination. FF: literature search. CM, MS, WLT, AE, and MH: literature screening. CM, WLT, JG, and TE: data extraction. CM, WLT, MS, AE, JG, TE, and MH: manuscript writing. All authors contributed to the article and approved the submitted version.

## Conflict of Interest

The authors declare that the research was conducted in the absence of any commercial or financial relationships that could be construed as a potential conflict of interest.
