# Supplementary material for: Fear of Cancer Recurrence, Health Anxiety, Worry, and Uncertainty: A Scoping Review About Their Conceptualization and Measurement Within Breast Cancer Survivorship Research
Source: Front Psychol. 2021 Apr 12;12:644932. doi: 10.3389/fpsyg.2021.644932 (PMC8072115; doi:10.3389/fpsyg.2021.644932)
Supplement: Supplementary file 1 [file Table_1.DOCX]

**Appendix A. Search Strategies**

**Medline**

| 1. ((cancer* or neoplas* or tumo?r* or malignan* or carcinoma* or adenocarcinoma* or choriocarcinoma* or sarcoma* or lymphoma* or melanoma* or myeloma* or oncolog*) adj5 (breast* or mammar*)).tw,kf. |  |
| --- | --- |
| 2. (DCIS or LCIS).tw,kf. |  |
| 3. ((ductal or lobular) adj3 in situ).tw,kf. |  |
| 4. breast neoplasms/ or breast carcinoma in situ/ or carcinoma, ductal, breast/ or carcinoma, lobular/ or "hereditary breast and ovarian cancer syndrome"/ or inflammatory breast neoplasms/ or triple negative breast neoplasms/ or unilateral breast neoplasms/ |  |
| 5. 1 or 2 or 3 or 4 |  |
| 6. ((fear or fears or anxiet* or anxious* or worry or worries or concern* or stress* or distress* or uncertainty or cope or coping or mood or depress* or wellbeing) adj11 (re?cur* or relaps* or progression or survivor*)).tw,kf. |  |
| 7. ((meaning or understand*) adj2 (life or existence)).tw,kf. |  |
| 8. distorted thinking.tw,kf. |  |
| 9. illness representation*.tw,kf. |  |
| 10. 7 or 8 or 9 |  |
| 11. (re?cur* or relaps* or progression).tw,kf. |  |
| 12. 10 and 11 |  |
| 13. Fear/ or Anxiety/ or Stress, Psychological/ or exp Adaptation, Psychological/ |  |
| 14. Recurrence/ or Neoplasm recurrence, Local/ |  |
| 15. 13 and 14 |  |
| 16. Neoplasm Recurrence, Local/px [Psychology] |  |
| 17. 6 or 12 or 15 or 16 |  |
| 18. 5 and 17 |  |
| 19. exp Breast NEOPLASMS/px [Psychology] |  |
| 20. Recurrence/ or Neoplasm recurrence, Local/ |  |
| 21. 19 and 20 |  |
| 22. 18 or 21 |  |

**Cinahl**

| S25 | S21 OR S24 |
| --- | --- |
| S24 | S22 AND S23 |
| S23 | (MH "Recurrence") OR (MH "Neoplasm Recurrence, Local") |
| S22 | (MH "Breast Neoplasms/PF") |
| S21 | S6 AND S20 |
| S20 | S7 OR S13 OR S18 OR S19 |
| S19 | (MH "Neoplasm Recurrence, Local/PF") |
| S18 | S16 AND S17 |
| S17 | (MH "Recurrence") OR (MH "Neoplasm Recurrence, Local") |
| S16 | S14 OR S15 |
| S15 | (MH "Stress, Psychological") OR (MH "Adaptation, Psychological") |
| S14 | (MH "Fear") OR (MH "Anxiety") OR (MH "Anticipatory Anxiety") OR (MH "Catastrophization") OR (MH "Suffering") OR (MH "Hopelessness") |
| S13 | S11 AND S12 |
| S12 | TI ((re?cur* or relaps* or progression) ) OR AB ((re?cur* or relaps* or progression)) |
| S11 | S8 OR S9 OR S10 |
| S10 | TI illness representation* OR AB illness representation* |
| S9 | TI distorted thinking OR AB distorted thinking |
| S8 | TI ((meaning or understand*) N2 (life or existence) ) OR AB ((meaning or understand*) N2 (life or existence)) |
| S7 | TI ((fear or fears or anxiet* or anxious* or worry or worries or concern* or stress* or distress* or uncertainty or cope or coping or mood or depress* or wellbeing) N11 (re?cur* or relaps* or progression or survivor*)) OR AB ((fear or fears or anxiet* or anxious* or worry or worries or concern* or stress* or distress* or uncertainty or cope or coping or mood or depress* or wellbeing) N11 (re?cur* or relaps* or progression or survivor*)) |
| S6 | S1 OR S2 OR S3 OR S4 OR S5 |
| S5 | (MH "Carcinoma, Lobular") |
| S4 | (MH "Breast Neoplasms") OR (MH "Carcinoma, Ductal, Breast") OR (MH "Hereditary Breast and Ovarian Cancer Syndrome") |
| S3 | TI ((ductal or lobular) N3 "in situ") OR AB ((ductal or lobular) N3 "in situ") |
| S2 | TI (DCIS or LCIS) OR AB (DCIS or LCIS) |
| S1 | TI ((cancer* or neoplas* or tumo?r* or malignan* or carcinoma* or adenocarcinoma* or choriocarcinoma* or sarcoma* or lymphoma* or melanoma* or myeloma* or oncolog*) N5 (breast* or mammar*) ) OR AB ( (cancer* or neoplas* or tumo?r* or malignan* or carcinoma* or adenocarcinoma* or choriocarcinoma* or sarcoma* or lymphoma* or melanoma* or myeloma* or oncolog*) N5 (breast* or mammar*)) |

**PsycInfo**

| 1. ((cancer* or neoplas* or tumo?r* or malignan* or carcinoma* or adenocarcinoma* or choriocarcinoma* or sarcoma* or lymphoma* or melanoma* or myeloma* or oncolog*) adj5 (breast* or mammar*)).tw. |  |
| --- | --- |
| 2. (DCIS or LCIS).tw. |  |
| 3. ((ductal or lobular) adj3 in situ).tw. |  |
| 4. Breast neoplasms/ |  |
| 5. 1 or 2 or 3 or 4 |  |
| 6. ((fear or fears or anxiet* or anxious* or worry or worries or concern* or stress* or distress* or uncertainty or cope or coping or mood or depress* or wellbeing) adj11 (re?cur* or relaps* or progression or survivor*)).tw. |  |
| 7. ((meaning or understand*) adj2 (life or existence)).tw. |  |
| 8. distorted thinking.tw. |  |
| 9. illness representation*.tw. |  |
| 10. 7 or 8 or 9 |  |
| 11. (re?cur* or relaps* or progression).tw. |  |
| 12. 10 and 11 |  |
| 13. exp Fear/ or Anxiety/ or Health anxiety/ or Panic/ or Psychological stress/ or Emotional adjustment/ |  |
| 14. "Relapse (Disorders)"/ |  |
| 15. 10 or 13 |  |
| 16. 11 or 14 |  |
| 17. 5 and 15 and 16 |  |
