# Supplementary material for: Fear of Cancer Recurrence, Health Anxiety, Worry, and Uncertainty: A Scoping Review About Their Conceptualization and Measurement Within Breast Cancer Survivorship Research
Source: Front Psychol. 2021 Apr 12;12:644932. doi: 10.3389/fpsyg.2021.644932 (PMC8072115; doi:10.3389/fpsyg.2021.644932)
Supplement: Supplementary file 2 [file Table_2.DOCX]

**Appendix B. Data Extraction Instrument**

**Article Number:**

| **Study Design** | |
| --- | --- |
| 1. Title |  |
| 2. Authors |  |
| 3. Year published |  |
| 4. Journal, Volume, Issue, Pages |  |
| 5. Types of Study / Study Design |  |
| **Study Population Details and Characteristics** | |
| 6. Study Purposes |  |
| 7. Settings |  |
| 8. Participant’s age (mean, range) |  |
| 9. Marital status |  |
| 10. Ethnicity |  |
| 11. % of BC patients and % of participants with  other types of cancers (for psychometric studies only) |  |
| 12. Stage of Cancer |  |
| 13. Time since the end of treatment/ diagnosis |  |
| **Details/Results Extracted from Source of Evidence** | |
| 14. Specify the construct(s) defined: FCR, W, U, HA  (If a clear definition is not given, specify the theoretical model/conceptualize model cited by the authors) |  |
| 15. Assessment tool/ measurements/ scales/  questionnaires used (if applicable) |  |
| 16. Specify if the measurement is self - reported,  clinician - reported, or otherwise (if applicable) |  |
| 17. Details of psychometric validation of tool  (if applicable) |  |
| 18. Characteristics related to the 4 constructs:  (as reported in the results/ discussion section) |  |
